# Ants’ navigation in an unfamiliar environment is influenced by their experience of a familiar route

**DOI:** 10.1038/s41598-017-14036-1

**Published:** 2017-10-26

**Authors:** Sebastian Schwarz, Antoine Wystrach, Ken Cheng

**Affiliations:** 1 School of Informatics, University of Edinburgh, 10 Crichton St, Edinburgh, EH8 9AB UK; 2 Centre de Recherches sur la Cognition Animale, CNRS, Université Paul Sabatier, Toulouse, F-31062 cedex 09 France; 3 Department of Biological Sciences, Macquarie University, Sydney, NSW 2109 Australia

## Abstract

When displaced experimentally from a food source (feeder) to unfamiliar terrain, ants run off a portion of the homeward vector or its entirety, depending on species and conditions, and then search systematically, turning in loops of ever increasing size. The Australian desert ant *Melophorus bagoti* runs off a smaller portion of its vector if the test site is more dissimilar to its nest area. Here we manipulated familiarity with the training route between a feeder and the ants’ nest to examine its effects when the ants were displaced to a distant site from the feeder. Naïve ants that arrived at an experimentally provided feeder for the first time were compared with experienced ants that had travelled the route for two days. At the unfamiliar test site, naïve ants ran off a longer portion of their vector from path integration than did experienced ants. Naïve ants also spread out in their systematic search slower than did experienced ants. We conclude that as ants learn the views encountered on their familiar route better, they identify more readily unfamiliar views. A scene distant from their nest area may not look as unfamiliar to a naïve ant as it does to an experienced ant.

## Introduction

Animals use a suite of strategies to navigate to a goal such as their home^1–3^. Prime strategies include the use of the surrounding scene, in various modalities, and path integration. In path integration, an animal keeps track of the distance and direction travelled on its outbound route to calculate a vector back to the starting point^4,5^. Both the use of the surrounding panorama and path integration are prone to errors. In natural conditions, this means often not quite getting exactly to a tiny desired goal. Animals back up these aforementioned strategies with systematic searching^6–8^. Most thoroughly studied in hymenopteran insects, in a systematic search, the insect makes loops around its start of search, with the loops increasing in size as the search wears on but occasionally returning back to the start^8^.

In desert ants, the subjects of our study, these navigational strategies work in parallel, and not one at a time following some decision akin to flipping a switch to a definite state^9^. Under a conflict between path integration and the surrounding panorama as to the direction home, for example, desert ants take an intermediate direction, species including the North African *Cataglyphis fortis* ^10^, the Australian *Melophorus bagoti* ^11^, and the European *Cataglyphis velox* ^12^. Vacillation between view based navigation and systematic searching, or perhaps their simultaneous operation, can also be observed in the meandering paths that desert ants sometimes take^13–16^. The ant seems to wiggle left and right even as it is heading in a definite direction according to its path integrator. *Melophorus bagoti* meanders more when the scene is unfamiliar, a measure of meander rising with increasing discrepancy between the views on their training routes and the views that they encounter on tests^14,15^. Such meandering has been interpreted as simultaneous deployment of systematic searching and path integration.

The conflict between path integration and systematic search is also reflected in the extent to which a vector from path integration is run off before the ant engages fully in search. The proportion of the vector that is run off depends on the species’ habitat—as opposed to phylogenetic lineage—based on the few species studied so far^13,16–19^. In *M. bagoti*, this proportion also depends on testing condition: the more unfamiliar or different the testing conditions are as compared with training conditions, the smaller the proportion of the vector that is run off before the ant begins searching^15,16,19,20^.

**Figure 1:**
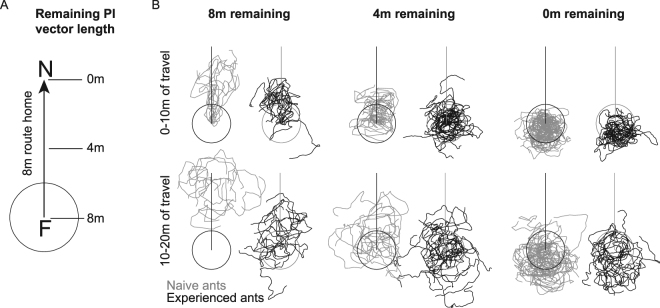
Schematic of experimental set up and 20 m of path from all ants tested. (**A**) Ants travelled a route between a feeder (F) 8 m from their nest (N) and their home. Experienced ants were taken for a test after 2 days of shuttling back and forth, while naïve ants were taken after their first arrival. Ants were taken either from the feeder (8 m condition, 8 m of vector remaining), half way on the route home (4 m condition, 4 m of vector remaining) or just before they entered their nest (0 m condition, 0 m of vector remaining). PI = path integration. (**B**) All paths run by the tested ants divided into the first 10 m of path length and the second 10 m of path length. A circle 2 m in radius has been drawn around each graph to help readers visualise the starting region on tests. No such circle was actually drawn at the test site.

In the current study, we explored in desert ants how learning a familiar route affects their navigational behaviour in a strange, unfamiliar terrain, by testing both well-trained and almost naïve red honey ants at a distant site never encountered by the ants before the test. Is such a site distinguished as unfamiliar from a naïve stage onwards or, more interestingly, does it require first familiarisation with the ant’s home environment for distant sites to be distinguished? To investigate this question, a feeder was set at a constant place 8 m from one *M. bagoti* nest (Fig. 1A). In a comparison of two regimes of training, homing ants were snatched from different stages of their training route from the feeder to their nest, transported to a distant test site, and released on the ground for a test. Many such displacement tests have been done in past studies, such as the studies already described above, on ants with various and sometimes unknown amount of experience of the route between the feeder and the nest. We, however, explicitly compared ants with different amount of experience on their familiar route, for the first time to our knowledge. One group of ants, called *experienced* ants, were tested two days after their first arrival at the feeder, while a second group, called *naïve* ants, were taken on their first visit to the feeder. The *naïve* ants were not totally naïve because *M. bagoti*, like many other species of ants (*Cataglyphis fortis* ^21,22^; *Ocymyrmex robustior* ^23^), scan and display learning walks around their nest before setting off on foraging excursions^24,25^. Most *M. bagoti* ants move up to 3 m from their nest on their learning walks. And foragers could also take a number of longer foraging excursions before arriving at an experimental feeder (personal observations). Would naïve ants behave differently on tests compared with experienced ants? Would experience of the environment around the nest affect the way path integration and systematic search are used in novel locations?

## Results

Ants—different individuals—were taken either a) from the feeder, b) halfway on their route home, or c) just before they entered their nest. In these different test conditions, the ants thus had approximately 8 m, 4 m, or 0 m of path-integrated vector left to run (Fig. 1A). On a test, a released ant would typically turn and scan in the vicinity of the release point, and then head off in a direction. Ants with any remaining vector from path integration tended to run some distance before meandering on what looked like searching behaviour (Fig. 1B). Their centre of search also appeared to be farther from the release point (higher on the *y*-axis). Zero-vector ants (0 m condition), both naïve and experienced, appeared to drift back, in the nest-to-feeder direction in the second 10 m of path (10–20 m path length, Fig. 1B).

### Path integration

**Figure 2:**
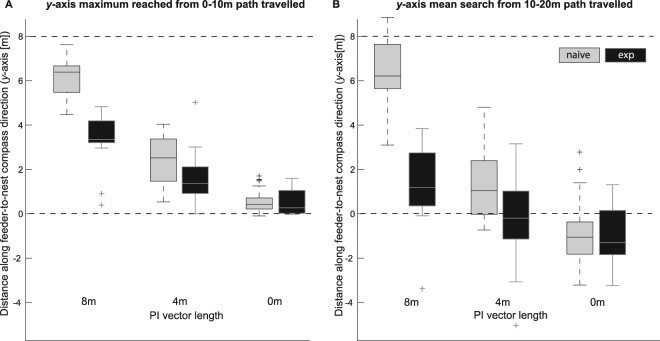
Proxy measures estimating extent of path integration (**A**) and centre of search (**B**). (**A**) The maximum value along the *y*-axis, which runs from the release point (0) in the feeder-to-nest direction, in the first 10 m of path length. (**B**) The mean value of all digitised points along the *y*-axis in the segments of paths from 10 m to 20 m of path length. The top dotted line indicates the *y* value of the fictive nest, where the nest would lie had the ants not been displaced for the test. The bottom dotted line indicates the *y* value of the release point. The boxes show the median in the centre line, and interquartile ranges at their top and bottom. The tails show values that are 1.5 times the distance from the median to each quartile. The + symbols show outlying values beyond the tails. PI = path integration.

In path integration, ants with a longer vector remaining, unsurprisingly, ran farther than ants with no vector remaining in the feeder-to-nest direction (along the *y*-axis on graphs in Fig. 2A). More interestingly, for ants with a remaining vector, naïve ants appeared to run farther than did experienced ants. Inferential statistics on the maximum *y* values in the first 10 m of path length, a proxy for the extent of path integration, confirms this. A two-way analysis of variance with group (naïve, experienced) and condition (8, 4, and 0 m) as factors showed significant main effects of both group (*F*_1,135_ = 59.5, *P* < 10^−11^) and condition (*F*_2,135_ = 229.1, *P* < 10^−43^). The analysis also found a significant interaction between group and condition (*F*_2,135_ = 23.3, *P* < 10^−8^). A priori contrasts between naïve and experienced ants were made for each test condition. We found significant differences between ants with different levels of experience for the 8 m test (*F*_1,135_ = 43.2, *P* < 10^−6^) and the 4 m test (*F*_1,135_ = 6.85, *P* = 0.012), but not for the 0 m test (*F*_1,135_ = 0.11).

### Centre of search

The centre of search seems to differ between conditions and experience in training (Fig. 2B). We estimated the centre of search in each ant as the mean *y* value of each ant in the second 10 m (path length 10 m to 20 m) of travel. Indeed, a two-way analysis of variance again showed main effects of group (*F*_1,135_ = 66.7, *P* < 10^–12^) and condition *F*_2,135_ = 97.3, *P* < 10^–26^). The analysis also found a significant interaction between group and condition (*F*_2,135_ = 26.3, *P* < 10^–9^). A priori contrasts between naïve and experienced ants were made for each test condition. We found significant differences between ants with different levels of experience for the 8 m test (*F*_1,135_ = 59.0, *P* < 10^–7^) and the 4 m test (*F*_1,135_ = 9.43, P = 0.004), but not for the 0 m test (*F*_1,135_ = 0.04).

### Search spread

**Figure 3:**
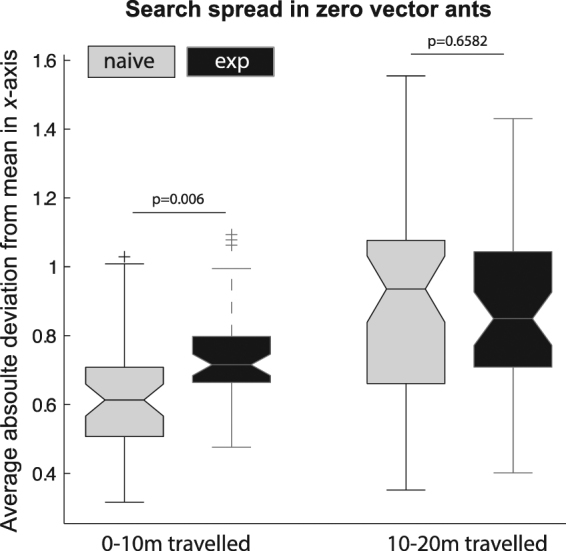
Proxy measure of search spread for zero-vector ants (0 m condition). The dependent measure was the mean absolute deviation of all digitised points along the *x*-axis, which is perpendicular to the feeder-to-nest direction. The measure is divided into the first 10 m of travel and the next 10 m of travel. The boxes show the median in the centre line, the interquartile ranges at their top and bottom, and the 95% confidence interval about the mean at their shoulders. The tails show values that are 1.5 times the distance from the median to each quartile. The + symbols show outlying values beyond the tails.

Differences between conditions in the spread of search are not apparent in Fig. 1B, but the search appears to spread out more from the first 10 m of path length to the second 10 m (path length 10–20 m), consistent with much past research on searching^7,8,26^. The spread of search was estimated by the average absolute deviation of *x* values from each ant’s path from its mean *x* value (Fig. 3). We analysed this variable for zero-vector ants, those in the 0 m condition, with group (naïve, experienced) as between-subjects factor and path segment (0–10 m, 10–20 m) as repeated measure. This analysis showed a significant main effect of path segment (*F*_1,66_ = 45.6, *P* < 10^–4^), but not of experience (*F*_1,66_ = 0.89). A significant interaction was also found (*F*_1,66_ = 4.60, *P* = 0.036). A priori contrasts showed that for the first 10 m of path segments, the two groups (experienced and naïve) differed significantly in spread along the *x*-axis (*F*_1,66_ = 8.07, *P* = 0.006), but that for path segments from 10–20 m of path length, the two groups did not differ significantly (*F*_1,66_ = 0.20). The search spread along the *x*-axis also increased in the 8 m and 4 m test conditions (Fig. S1), but we have not analysed those data statistically.

## Discussion

To highlight our results, we displaced ants from a feeder, or from their route between the feeder and their nest after they had travelled half way, or all the way home, and released them at a distant unfamiliar site for a single test. We compared ants that had visited the feeder for two days (experienced ants) with less experienced ants that arrived at the feeder for the first time (naïve ants). For ants in test conditions with some path-integrated vector left to run (full-vector ants displaced from the feeder or half-vector ants displaced from the half-way point), naïve ants ran farther than did experienced ants. These naïve ants’ centre of search was also farther along the fictive route home than that of experienced ants. In zero-vector ants, which had run off their vector, some evidence suggests that experienced ants spread out their search faster than naïve ants. The two groups’ search spread differed significantly in the first 10 m of path length, but not in the second 10 m of path length.

At an unfamiliar test site, the use of familiar terrestrial panoramic cues for guidance is not possible. The tested ants must have relied on a combination of path integration and systematic searching. As introduced already, in our test species, *Melophorus bagoti*, tests with full-vector ants show that the more unfamiliar the surrounding scene at the test site, the less the distance that they follow their path-integrated vector before engaging in systematic searching^13,15,16,19,20^. The proportions of vector trotted off on average span from ~40% to ~90% in different conditions. The highest proportions came on tests when two experimentally provided rows of cylinders traversing the entire route from feeder to nest were reproduced at the test site. These studies defined the proportion of vector run off by identifying the point at which the homing ant first made a sharp turn, with the definition of a sharp turn varying across studies. We used the maximum extent of travel in the first 10 m of path length. Our estimate likely provides a higher estimate of the proportion of vector run off than those of previous studies. Keeping this caveat in mind, our key results show that 8 m naïve ants in this study scored ~80% of vector run off, while 8 m experienced ants scored ~40%. These differences between naïve and experienced ants suggest that what counts as unfamiliar depends on the ants’ experience. Desert ants need to learn what is familiar in the environment around their nest^22,23,27^, and the better they learn their familiar environment, the more readily they can distinguish an unfamiliar scene from all the familiar scenes that they had encountered.

The kernel of an explanation for such results is pattern separation. In pattern separation, the brain’s representations of similar entities, of which visual scenes are only one instance, are moved apart in representational space as the animal learns. Such an idea has been proposed as a mechanism for insect learning in other contexts^28–33^ and we are suggesting that it applies to this study as well. Scene learning in ants is likely subserved by the mushroom bodies^32^, which typically modulate the valence of sensory inputs by shifting the relative balance within an ensemble of both appetitive and aversive output pathways^31,34^. We suggest that in learning a scene, the relevant circuit—likely the ensemble of mushroom body output neurons^32^ —that responds more positively to the familiar scene also responds more negatively to unfamiliar scenes.

With the interpretation of the distance of the integrated path (vector) run (Fig. 2A) at hand, the key results on the centre of search follow readily as a consequence. The centre of search (Fig. 2B) reflects a similar pattern because to a first approximation, search is typically centred at the point where the ant stops following path integration. The qualificatory language reflects our current understanding that an ant does not decide at some precise point to switch definitively from path integration to systematic search. Very likely, these strategies are in operation simultaneously, with the weight shifting away from path integration as the navigator runs down the vector^12^. Several models now invoke the simultaneous operation of various routines, with the weights assigned to the routines being flexible^9,10,35,36^. As such, we do not put much weight on possible differences in mean values between corresponding conditions in Fig. 2A and B. Figure 2A shows maximum *y* values, while Fig. 2B shows mean *y* values, with Fig. 2A probably having a combination of path integration and searching contributing to its values.

One other intriguing characteristic found in Fig. 2B is that for ants with some vector remaining, the centres of search show no tendency to drift towards 8 m on the *y*-axis for the 8 m group, or towards 4 m for the 4 m group, where the global vector would drive the ants in those conditions. Something seems to be stopping the ants from running off the global vector during search. In contrast, *Cataglyphis* ants (*C. albicans* and *C. bicolor*) in Wehner and Srinivasan’s^6^ study centred their searches at the ends of their global-vector runs. Path integration during the search process pulls *Cataglyphis* ants back to the starting point of search occasionally^6,37^. In our study, however, the question arises as to what is pulling the ants back to the starting point of search, a characteristic of area-restricted search notable in Fig. 1B. We can come up with two types of explanations. The first hypothesis is that some new vector, which can be considered a negative vector to the global vector, serves to annul in part or subtract a portion of the global vector. Adding such a vector would mean that path integration, now taking in tow both the global vector and the backtracking vector, would bring the searching ant occasionally back to a centre of search that is somewhere between the release point on a test and the fictive nest^38^. The second hypothesis is that path integration has been reset, such that its zero state is no longer at the fictive nest, but is located between the start of the run and the fictive nest. These explanations of course require further experimental evidence to affirm or deny them.

One point of note about the centres of search are the negative mean values for zero-vector ants, both naïve and experienced. This reflects a small amount of back-tracking, or heading opposite to the feeder-to-nest direction. This pattern of results has been found before for zero-vector ants that had travelled a substantial distance (almost) home before they were displaced^36^. This tendency may be functional in that ants that are displaced near home to unfamiliar terrain have likely gone beyond their nest, where the terrain, having hardly been travelled, would look less familiar. Such mishaps could take place in Central Australia with occasional wind gusts.

Figure 3 shows that zero-vector experienced ants seem to spread their search out faster than zero-vector naïve ants, although by 10–20 m of search path, the spread was similar in the two groups. Also, such a difference is only apparent in the zero-vector ants (0 m condition), with the search patterns of naïve and experienced ants being similar in the 4 m and 8 m conditions (Fig. S1). If the difference in searching between naïve and experienced ants is real, we can attribute it to the experienced ants distinguishing more readily the unfamiliar scene from their familiar scenes. Ants of this species searching in unfamiliar terrain spread their search out quicker when the surrounding scenery is unfamiliar^19,39^ than when the scene looks familiar^19,26^.

Finally, in all conditions of testing, the search spreads out as it goes on (Fig. 3). This result unsurprisingly replicates a common pattern found in hymenopteran searching in this species^19,26,39,40^ and in others (*C. bicolor* and *C. albicans* ^6^; *C. fortis* ^37^; *Melophorus* sp., a species that has not been named as yet^19^; honeybees, *Apis mellifera* ^41,42^:).

## Conclusions

This study found differences between naïve and experienced ants when they were displaced to a distant site to integrate a path and engage in systematic search. Naïve ants were first-time arrivers at the feeder from which they were displaced, and hence less experienced with the route between their home and the feeder. When released on unfamiliar terrain, experienced ants ran less far in integrating a path than did naïve ants, they searched earlier, and they might also have spread their systematic search out faster, as if experienced ants had perceived the distant site as more unfamiliar than naïve ants did. The results suggest that as the familiar scenes become more familiar, unfamiliar scenes seem more different from the familiar scenes, possibly as a result of pattern separation.

## Methods

### Animals

Red honey ants (*Melophorus bagoti* Lubbock) are found in a wide range of Central Australia. The ants forage individually in the hotter part of the day during the southern summer months^24,43^. Foragers from one nest at a private property on the outskirts of Alice Springs, Australia, ~10 km south of town centre, took part in the experiment. Australia does not have ethical requirements concerning work with ants, but the experimental procedures were completely non-invasive and, as in previous behavioural research, had no notable adverse effects on the individuals and on the colony.

### Set up and setting

A plastic feeder (~15 × 15 × 9 cm deep) was set up 8 m from the nest entrance. The feeder was sunk into the ground with its lip at ground level and provisioned with food, crumbs of cookie (Arnott™ brand) and pieces of mealworms. Different ants prefer one or the other kind of food, and an individual ant might vary the menu item that it grabs across multiple trips. The feeder functioned as a trap as well because its slippery sides were practically impossible for the ants to climb. Foraging ants were free to drop into the feeder.

The environment contained mostly open space, with a few low buildings scattered in it amidst eucalypt trees and bushes (*Hakea* and *Acacia* species). The invasive buffel grass (*Cenchrus ciliaris*) dominated the vegetation. In the distance to the north, the McDonnell ranges were visible. A distinct visual surround delineated the panorama.

At a distant site with an unfamiliar view to the ants, a gridded test field was set up. A large grid of 1-m squares was made by sticking hooked tent pegs into the ground at the vertices of the grid, and then winding string around the pegs. The grid was thus readily visible to an experimenter, but hardly interfered with the travel of ants being tested.

### Procedure

Ants were divided into two groups, with each group tested under three conditions, making a 2 × 3 factorial design. Ants in the naïve group were tested upon their first arrival at the feeder. Ants in the experienced group were marked with a single colour (Tamiya™ brand acrylic paint) that coded the day of arrival and the ant’s start of training. Experienced ants were tested two days after their day of arrival. During training, painted ants were allowed to run home with food after each visit to the feeder; they were lifted out of the feeder after they had grabbed a piece of food, after which they inevitably trotted home.

Each ant was tested only once, making all data between-subjects. Each naïve and experienced ant was tested in one of three different conditions. In the 8 m condition (27 experienced ants, 18 naïve ants), an ant was taken from the feeder to the test site; these ants thus had a full 8 m of homing vector from path integration. In the 4 m condition (32 experienced ants, 21 naïve ants), an ant was lifted from the feeder and allowed to run ~4 m, halfway home. It was then captured and taken for a test; these ants thus had a ~4 m vector remaining. In the 0 m condition (23 experienced ants, 48 naïve ants), an ant was lifted from the feeder and allowed to run the full 8 m home. Just before it entered the nest, it was captured and taken for a test; these ants thus had no vector remaining and are traditionally called zero-vector ants (see Fig. 1A).

On a test, the captured ant was placed in a darkened tube and taken to the test field. After ensuring that the ant still held on its piece of food, the ant was released at one of the vertices of the grid. Ants that dropped their food were not tested. On a piece of gridded paper, the path of the ant was plotted, for long enough to ensure that at least 20 m of path length had been obtained.

### Data analysis

The paths on paper were digitised using GraphClick™ (www. arizona-software.ch/graphclick). In the coordinate system, the *y*-axis corresponds to the feeder-to-nest direction, increasing towards the nest. The *x*-axis then corresponds to sideways travel with respect to the direction to the nest. The first 20 m of path length were used for analysis.

Past research on path integration in this and other ants has typically defined the end of path integration by some criterion characterizing a sharp turn. This was inappropriate for the current study because 1) zero-vector ants might be expected to start searching immediately, and 2) this species has been shown to mix path integration and search^9,44^, making it hard to separate the end of path integration from the start of search. We thus opted to use an objective measure without invoking any notion of a sharp turn: the maximum *y* value for the first 10 m of path length of each individual served as a proxy for extent of travel based on path integration.

To estimate the centre of search along the *y* axis (in the feeder-to-nest direction), the next 10 m of path, path length from 10 m to 20 m, was used. We took the mean *y* value of this second path section as a proxy for the centre of search for each individual.

Finally, to estimate the spread of search, we used the *x*-axis values. As any path integration or backtracking^36^ should move a navigating ant mostly in the feeder-to-nest direction (along the *y*-axis), *x* values are far less contaminated by path integration than are *y* values. Spread along the *x*-axis thus better reflects search spread than spread along the *y*-axis. As proxy for search spread, we used the average absolute deviation of *x* values from the mean *x* value for each individual, separately for the first 10 m of path length, and for the path segment from 10 m to 20 m of path length. Thus, the mean *x* value was calculated, and the difference between each *x* value and the mean computed; all these differences were turned positive (absolute values) and the mean of these absolute differences estimated search spread. We examined only zero-vector ants (0 m condition) because these ants are not expected to be engaging in any path integration, a process that could distort our attempt to isolate the search pattern.

As all these dependent variables were calculated for each ant’s path separately, and not for all paths together, measures from individuals could then be entered into standard descriptive and parametric inferential statistics. For the maximum *y* value in the first 10 m of search path and the mean *y* value in the search path from 10–20 m of path length, we further compared naïve vs. experienced ants in each of the test conditions with a priori contrasts, as we predicted possible differences in full-vector (8 m) and half-vector (4 m) ants, but not zero-vector (0 m) ants. The 8 m naïve ants were contrasted against the 8 m experienced ants, the 4 m naïve ants were contrasted against the 4 m experienced ants, and the 0 m naïve ants were contrasted against the 0 m experienced ants.

### Data availability

All data generated or analysed during this study are included in this published article (and its Supplementary Information files).

## Electronic supplementary material

Supplementary Figure S1

Explanation of raw data

raw data: full set

## References

[1] Cheng, K. & Jeffery, K. J. in *APA handbook of comparative psychology* Vol. 2: Perception, Learning, and Cognition (ed J. Call) 463–483 (American Psychological Association, 2017).

[2] Pritchard, D. J. & Healy, S. D. in *APA handbook of comparative psychology* Vol. 2: Perception, Learning, and Cognition (ed J. Call) 485–508 (American Psychological Association, 2017).

[3] Graham, P. & Wystrach, A. in *Animal cognition: Principles, evolution, and development *(ed M. C. Olmstead) Chapter 4 (Nova Science, 2017).

[4] Collett M, Collett TS (2000). How do insects use path integration for their navigation?. Biological Cybernetics.

[5] Wehner, R. & Srinivasan, M. V. in *Th*e *neurobiology of**spatial behaviour* (ed K.J. Jeffery) 9–30 (Oxford University Press, 2003).

[6] Wehner R, Srinivasan MV (1981). Searching behaviour of desert ants, genus *Cataglyphis* (Formicidae, Hymenoptera). Journal of Comparative Physiology A.

[7] Merkle T, Knaden M, Wehner R (2006). Uncertainty about nest position influences systematic search strategies in desert ants. Journal of Experimental Biology.

[8] Schultheiss P, Cheng K, Reynolds AM (2015). Searching behavior in social Hymenoptera. Learning and Motivation.

[9] Wehner R, Hoinville T, Cruse H, Cheng K (2016). Steering intermediate courses: desert ants combine information from various navigational routines. Journal of Comparative Physiology A.

[10] Collett M (2012). How navigational guidance systems are combined in a desert ant. Current Biology.

[11] Legge ELG, Wystrach A, Spetch ML, Cheng K (2014). Combining sky and earth: desert ants (*Melophorus bagoti*) show weighted integration of celestial and terrestrial cues. Journal of Experimental Biology.

[12] Wystrach A, Mangan M, Webb B (2015). Optimal cue integration in ants. Proceedings of the Royal Society B-Biological Sciences.

[13] Bühlmann C, Cheng K, Wehner R (2011). Vector-based and landmark-guided navigation in desert ants inhabiting landmark-free and landmark-rich environments. Journal of Experimental Biology.

[14] Wystrach A, Schwarz S, Schultheiss P, Beugnon G, Cheng K (2011). Views, landmarks, and routes: how do desert ants negotiate an obstacle course?. Journal of Comparative Physiology A.

[15] Cheng K, Middleton EJT, Wehner R (2012). Vector-based and landmark-guided navigation in desert ants of the same species inhabiting landmark-free and landmark-rich environments. Journal of Experimental Biology.

[16] Cheng K, Schultheiss P, Schwarz S, Wystrach A, Wehner R (2014). Beginnings of a synthetic approach to desert ant navigation. Behavioural Processes.

[17] Beugnon G, Lachaud J-P, Chagné P (2005). Use of long-term stored vector information in the neotropical ant *Gigantiops destructor*. Journal of Insect Behavior.

[18] Schultheiss P, Schwarz S, Cheng K, Wehner R (2012). Foraging ecology of an Australian salt-pan desert ant (genus *Melophorus*). Australian Journal of Zoology.

[19] Schultheiss P (2016). Similarities and differences in path integration and search in two species of desert ants inhabiting a visually rich and a visually barren habitat. Behavioral Ecology and Sociobiology.

[20] Narendra A (2007). Homing strategies of the Australian desert ant *Melophorus bagoti* I. Proportional path integration takes the ant half-way home. Journal of Experimental Biology.

[21] Wehner R, Meier C, Zollikofer C (2004). The ontogeny of foraging behaviour in desert ants. Cataglyphis bicolor. Ecological Entomology.

[22] Fleischmann PN, Christian M, Müller VL, Rössler W, Wehner R (2016). Ontogeny of learning walks and the acquisition of landmark information in desert ants. Cataglyphis fortis. Journal of Experimental Biology.

[23] Müller M, Wehner R (2010). Path integration provides a scaffold for landmark learning in desert ants. Current Biology.

[24] Muser B, Sommer S, Wolf H, Wehner R (2005). Foraging ecology of the thermophilic Australian desert ant. Melophorus bagoti. Australian Journal of Zoology.

[25] Wystrach A, Philippides A, Aurejac A, Cheng K, Graham P (2014). Visual scanning behaviours and their role in the navigation of the Australian desert ant *Melophorus bagoti*. Journal of Comparative Physiology A.

[26] Schultheiss P, Wystrach A, Legge ELG, Cheng K (2013). Information content of visual scenes influences systematic search of desert ants. Journal of Experimental Biology.

[27] Wystrach A, Beugnon G, Cheng K (2012). Ants might use different view-matching strategies on and off the route. Journal of Experimental Biology.

[28] Bazhenov M, Huerta R, Smith BH (2013). A computational framework for understanding decision making through integration of basic learning rules. The Journal of Neuroscience.

[29] Perry, C. J., Barron, A. B. & Cheng, K. Invertebrate learning and cognition: relating phenomena to neural substrate. *Wiley Interdisciplinary Reviews: Cognitive Science* doi: 10.1002/wcs.1248 (2013). 10.1002/wcs.1248 26304245

[30] Andrew SC (2014). Peak shift in honey bee olfactory learning. Animal Cognition.

[31] Cohn R, Morantte I, Ruta V (2015). Coordinated and compartmentalized neuromodulation shapes sensory processing in *Drosophila*. Cell.

[32] Webb B, Wystrach A (2016). Neural mechanisms of insect navigation. Current Opinion in Insect Science.

[33] Peng F, Chittka L (2017). A simple computational model of the bee mushroom body can explain seemingly complex forms of olfactory learning and memory. Current Biology.

[34] Aso Y (2014). Mushroom body output neurons encode valence and guide memory-based action selection in Drosophila. eLife.

[35] Cruse H, Wehner R (2011). No need for a cognitive map: Decentralized memory for insect navigation. PLoS Computational Biology.

[36] Wystrach A, Schwarz S, Baniel A, Cheng K (2013). Backtracking behaviour in lost ants: an additional strategy in their navigational toolkit. Proceedings of the Royal Society B-Biological Sciences.

[37] Müller M, Wehner R (1994). The hidden spiral: systematic search and path integration in desert ants, *Cataglyphis fortis*. Journal of Comparative Physiology A.

[38] Goldschmidt, D., Manoonpong, P. & Dasgupta, S. A neurocomputational model of goal-directed navigation in insect-inspired artificial agents. *Frontiers in Neurorobotics* **11**, 20, https://doi.org/10.3389/fnbot.2017.00020 (2017). 10.3389/fnbot.2017.00020 PMC5388780 28446872

[39] Schultheiss P, Cheng K (2011). Finding the nest: inbound searching behaviour in the Australian desert ant. Melophorus bagoti. Animal Behaviour.

[40] Schultheiss P, Cheng K (2013). Finding food: outbound searching behavior in the Australian desert ant *Melophorus bagoti*. Behavioral Ecology.

[41] Reynolds AM (2007). Displaced honey bees perform optimal scale-free search flights. Ecology.

[42] Reynolds AM, Smith AD, Reynolds DR, Carreck NL, Osborne JL (2007). Honeybees perform optimal scale-free searching flights when attempting to locate a food source. Journal of Experimental Biology.

[43] Schultheiss P, Nooten SS (2013). Foraging patterns and strategies in an Australian desert ant. Austral Ecology.

[44] Narendra A (2007). Homing strategies of the Australian desert ant *Melophorus bagoti* II. Interaction of the path integrator with visual cue information. Journal of Experimental Biology.

