# Supplementary material for: Ants’ navigation in an unfamiliar environment is influenced by their experience of a familiar route
Source: Sci Rep. 2017 Oct 26;7:14161. doi: 10.1038/s41598-017-14036-1 (PMC5658437; doi:10.1038/s41598-017-14036-1)
Supplement: Supplementary file 1 — Supplementary Figure S1 [file 41598_2017_14036_MOESM1_ESM.pdf]

Ants' navigation in an unfamiliar environment is influenced by their experience of a familiar route

Sebastian Schwarz, Antoine Wystrach, Ken Cheng

Supplementary Figure 1

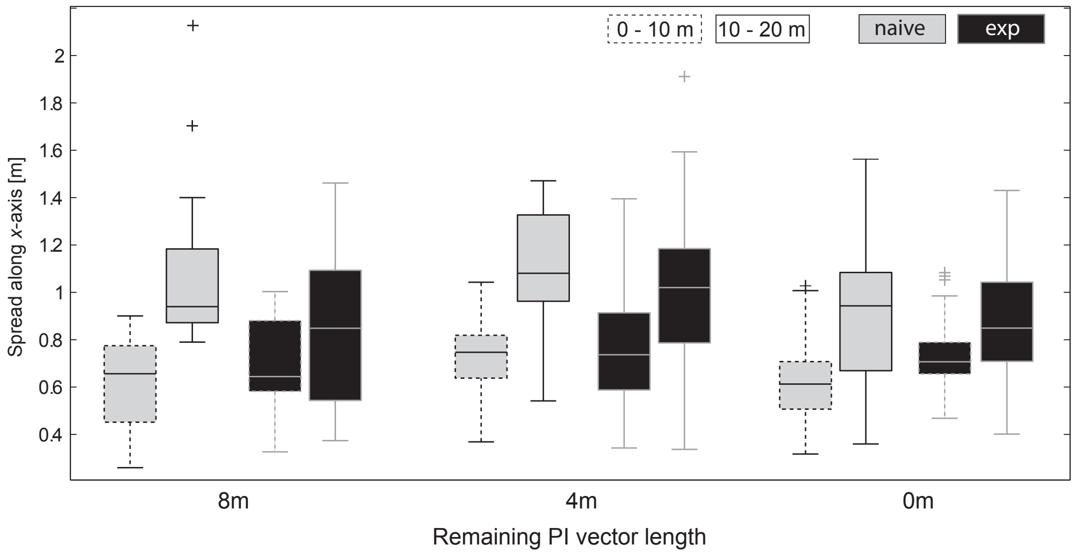

Figure S1. Proxy measure of search spread for all ants. The dependent measure was the mean absolute deviation of all digitised points along the *x*-axis, which is perpendicular to the feeder-to-nest direction. The measure is divided into the first 10 m of travel and the next 10 m of travel. The boxes show the median in the centre line, and the interquartile ranges at their top and bottom. The tails show values that are 1.5 times the distance from the median to each quartile. The + symbols show outlying values beyond the tails (PI = path integration).
