# Supplementary material for: Ants’ navigation in an unfamiliar environment is influenced by their experience of a familiar route
Source: Sci Rep. 2017 Oct 26;7:14161. doi: 10.1038/s41598-017-14036-1 (PMC5658437; doi:10.1038/s41598-017-14036-1)
Supplement: Supplementary file 2 — Explanation of raw data [file 41598_2017_14036_MOESM2_ESM.doc]

Paths as XY data digitised by clicking regularly along the individuals' path recorded by hand.

- Conditions names indicate:

FV=Full Vector (8m remaining PI vector)

HV=Half Vector (4m remaining PI vector)

ZV=Zero Vector (0m remaining PI vector)

naive/exp = naive or experienced groups.

- Ant_id= individual ant id (each ant is tested only once), the id are independant across conditions.

1st line = header

2d line till last line = points'coordinates sampled sequentially along the path.

scale: meters.

Release point's coordinates = (0,0).

Feder-to-nest compass direction along the positive Y axis.
